# Deep clustering of bacterial tree images

**DOI:** 10.1098/rstb.2021.0231

**Published:** 2022-10-10

**Authors:** Maryam Hayati, Leonid Chindelevitch, David Aanensen, Caroline Colijn

**Affiliations:** ^1^ School of Computing Science, Simon Fraser University, Burnaby, British Columbia, Canada V5A 1S6; ^2^ Department of Mathematics, Simon Fraser University, Burnaby, British Columbia, Canada V5A 1S6; ^3^ Department of Infectious Disease Epidemiology, Imperial College, Praed Street, London W2 1NY, UK; ^4^ Big Data Institute, University of Oxford, Old Road Campus, Oxford OX3 7LF, UK

**Keywords:** phylogenetics, clustering, deep learning, image processing

## Abstract

The field of genomic epidemiology is rapidly growing as many jurisdictions begin to deploy whole-genome sequencing (WGS) in their national or regional pathogen surveillance programmes. WGS data offer a rich view of the shared ancestry of a set of taxa, typically visualized with phylogenetic trees illustrating the clusters or subtypes present in a group of taxa, their relatedness and the extent of diversification within and between them. When methicillin-resistant *Staphylococcus aureus* (MRSA) arose and disseminated widely, phylogenetic trees of MRSA-containing types of *S. aureus* had a distinctive ‘comet’ shape, with a ‘comet head’ of recently adapted drug-resistant isolates in the context of a ‘comet tail’ that was predominantly drug-sensitive. Placing an *S. aureus* isolate in the context of such a ‘comet’ helped public health laboratories interpret local data within the broader setting of *S. aureus* evolution. In this work, we ask what other tree shapes, analogous to the MRSA comet, are present in bacterial WGS datasets. We extract trees from large bacterial genomic datasets, visualize them as images and cluster the images. We find nine major groups of tree images, including the ‘comets’, star-like phylogenies, ‘barbell’ phylogenies and other shapes, and comment on the evolutionary and epidemiological stories these shapes might illustrate.

This article is part of a discussion meeting issue ‘Genomic population structures of microbial pathogens’.

## 1. Introduction

Genomic epidemiology is increasingly used as a toolkit in public health surveillance. In many jurisdictions, public health laboratories gather large numbers of bacterial isolates and perform whole-genome sequencing (WGS) to monitor for antibiotic resistance, detect recent transmission clusters, determine the nature and extent of introductions from outside their community, and monitor bacterial populations for newly emerging resistance. These datasets provide a rich view of the ongoing diversification, evolution and selection in pathogens. However, the interpretation of WGS data for public health can be challenging. For example, while molecular typing schemes (VNTR [1], MIRU-VNTR [2], spoligotyping [3], RFLP [4], PFGE [5] and RAPD-PCR [6]) typically offer a clear ‘in or out’ signal for whether a new isolate is in a defined cluster or not, WGS data may have no established cutoff that performs well at grouping isolates together or denoting them as distinct.

WGS data do offer a detailed view of the relatedness among a set of isolates, typically seen through the lens of a phylogenetic tree. When a local isolate can be placed on a phylogenetic tree containing previous isolates from the same area and also isolates from the global population of the pathogen, this can build perspective and offer useful information for public health. For example, phylogeographic tools may be used to infer the origin of a locally circulating strain, if sequencing in the source region is sufficiently comprehensive and global databases contain representative information [7,8]. Placing isolates on a tree and annotating the tree with antibiotic resistance phenotypes help visualize the evolution and spread of resistance and can help determine whether resistance is predominantly acquired de novo or transmitted from person to person.

The proximity of an isolate or group of interest to other annotated clusters, the relative frequency and placement of resistance, the time and location when resistance was acquired in the tree and the extent of geographical mixing are examples of features that WGS data, annotation and phylogenetic trees can help us understand [9–11].

Methicillin-resistant *Staphylococcus aureus* (MRSA) is a major cause of hospital-acquired infections that are becoming increasingly difficult to combat because of emerging resistance to all current antibiotic classes [12]. When MRSA first emerged in the UK Midlands it was rapidly geographically disseminated, and it diversified rapidly as well [13]. The rapid emergence of methicillin-resistant isolates resulted in a distinct comet-like phylogenetic tree (when visualized in a radial way), which has two distinct parts. The comet ‘head’ represents the recent rapid expansion of MRSA isolates, and has a ‘burst’ of divergent lineages with a common origin. The ‘tail’ reflects the predominantly drug-sensitive isolates (MSSA) [14], which are more genetically diverse (as characterized by longer branches, both immediately ancestral to the tips and within the clades in the tail).

More broadly, the shape of a phylogenetic tree is known to contain information about the process that created the tree [15,16]. In this context, the phrase ‘tree shape’ is usually used to denote the connectivity structure of the tree, in the sense of a graph: nodes and edges (often without considering the lengths of the branches). Key summary measures of tree shape include measures of asymmetry (Colless [17] or Sackin [18] imbalance), the numbers of ‘cherries’ (two tips joined by their common parent in the tree), and so on. By contrast, in the MRSA example, the *image* of the phylogeny has helped researchers to understand the process of MRSA emergence and how it is reflected in the patterns of relatedness among *S. aureus* taxa. The tree image reflects the edge connectivity, the branch lengths and the choice of layout algorithm. The MRSA example illustrates one prominent case in which the image of the tree has a clear interpretation. Anecdotally, researchers use and interpret such tree images, often with metadata annotated as colour on the trees, in addition to using trees to group taxa into sub-populations and other tasks. However, there is not a clear taxonomy of the different sorts of images that arise in bacterial whole-genome datasets.

Here, we take the first steps towards such a taxonomy. In particular, we seek to determine whether there are clear recognizable tree images, analogous to the ‘comet-like’ phylogenies that illustrate MRSA emergence, in other bacterial pathogens and other settings of *S. aureus*. These characteristic images might signify shared epidemiology, ecology or evolution, and like the comet, provide a set of reference tree shapes to aid public health laboratories in placing new isolates in context. To this aim, we collected a set of bacterial trees from the NCBI Pathogen Detection Pipeline [19] and built a set of tree images from the subtrees of the original trees. We use convolutional neural networks and a consensus approach to clustering to explore tree shapes in this large dataset.

## 2. Methods

We cluster a collection of images derived from bacterial phylogenetic trees. Our method proceeds according to the following stages. We begin by applying a deep learning-based dimensionality reduction method to extract the important features of the images. We then apply three clustering algorithms (*K*-means clustering, hierarchical clustering, and affinity propagation) on reduced-size representations of the images. Lastly, we ensemble the three clustering algorithms to identify groups of tree images that are present in the data.

### (a) Data preprocessing and data augmentation

We use phylogenetic trees from the NCBI Pathogen Detection Pipeline [19] as this is a large, publicly available repository of sequence data where phylogenetic trees are available. We downloaded the trees in 2018. This dataset incorporates bacterial pathogen genomic sequences originating in food, environmental sources and human hosts. The bacterial pathogen genera or species present in the dataset are summarized in table 1.

**Table 1.** Phylogenetic trees downloaded from NCBI Pathogen Detection Pipeline. We extracted all the subtrees of size 35–100 from each tree. The second column shows the size of each tree, while the third and the fourth columns state the number of extracted subtrees and the number of non-overlapping subtrees, respectively.

species	no. tips	no. subtrees	non-overlapping
*Acinetobacter*	1646	75	14
*Campylobacter*	9180	406	106
*Citrobacter_freundii*	241	3	1
*Clostridioides_difficile*	550	24	7
*Clostridium_botulinum*	163	4	1
*Clostridium_perfringens*	191	7	2
*Corynebacterium_striatum*	46	1	1
*Cronobacter*	349	10	6
*Elizabethkingia*	157	15	2
*Enterobacter*	1524	86	16
*Escherichia_coli_Shigella*	35 063	1838	416
*Klebsiella*	5812	271	61
*Klebsiella_oxytoca*	273	14	3
*Kluyvera_intermedia*	15	0	0
*Legionella_pneumophila*	295	9	4
*Listeria*	7253	411	78
*Morganella*	72	6	1
*Mycobacterium_tuberculosis*	2844	121	32
*Neisseria*	1890	103	23
*Photobacterium_damselae*	42	1	1
*Providencia*	156	7	1
*Pseudomonas_aeruginosa*	2789	144	35
*Salmonella*	34 939	1965	403
*Serratia*	273	16	4
*Staphylococcus_pseudintermedius*	377	13	3
*Vibrio_cholerae*	569	26	5
*Vibrio_parahaemolyticus*	740	28	8
*Vibrio_vulnificus*	245	22	2

We extracted two sets of subtrees for training and clustering. The first set contains all the subtrees of sizes 35–100, and is obtained by traversing all the subtrees of a large tree (defined by the subtrees’ roots, which are the internal nodes of the tree) in a pre-order traversal, and keeping those subtrees whose size falls within the accepted range. We chose this size range largely for pragmatic reasons, as it allows a sufficient number of trees to be obtained (particularly where we seek non-overlapping trees) given the data available, yet 35–100 taxa are sufficient to illustrate patterns of evolution like the MRSA ‘comet’ (with a head, a tail, and distinct patterns of relatedness in each). The second set contains a non-overlapping collection of these subtrees obtained by the following greedy algorithm: We iteratively traverse the set of all subtrees of an appropriate size, listed in pre-order, from left to right, and construct a final list of non-overlapping subtrees starting from an empty list. At each iteration, the node under consideration gets appended to the final list if and only if none of its ancestors appears in the final list.

All the subtrees are saved as images of size 160×160 pixels in the portable network graphics (PNG) format. We use a radial layout algorithm, originally implemented in PHYLIP [20] and subsequently implemented in other algorithms. The radial layout gives each subtree an angular space that is proportional to the number of tips in it. It iterates through internal nodes of the tree, allocating the (angular) space for a subtree among its descendant subtrees. This approach places closely related tips very close together in the image. This is an advantage in our context, because it means that the image is relatively robust to instances where many very similar tips are sequenced (which could occur in an outbreak investigation, for example).

This results in two sets of images, with sizes 5626 (overlapping) and 1254 (non-overlapping), respectively. We used a data augmentation technique and added the rotation of each image by 45*n* degrees for 1 ≤ *n* ≤ 7 (i.e. all the multiples of one-eighth of a turn). This procedure results in 45 008 images (from overlapping trees) and 10 032 images (from non-overlapping trees), respectively. These images were used to train the auto-encoder, to obtain a low-dimensional faithful representation of the images. We note that because our auto-encoder is not doing classification, we did not train the network to classify rotated versions of an image as the same class (rather, we did clustering, in a two-stage approach that does not lend itself to including a condition that pre-specified images cluster together in its optimization. Furthermore, the layout algorithm is not rotation-invariant). We therefore used auto-augmentation primarily to obtain more images on which to train an auto-encoder that generates a faithful reproduction of its input image.

### (b) Dimensionality reduction with convolutional auto-encoders

**Figure 1.**
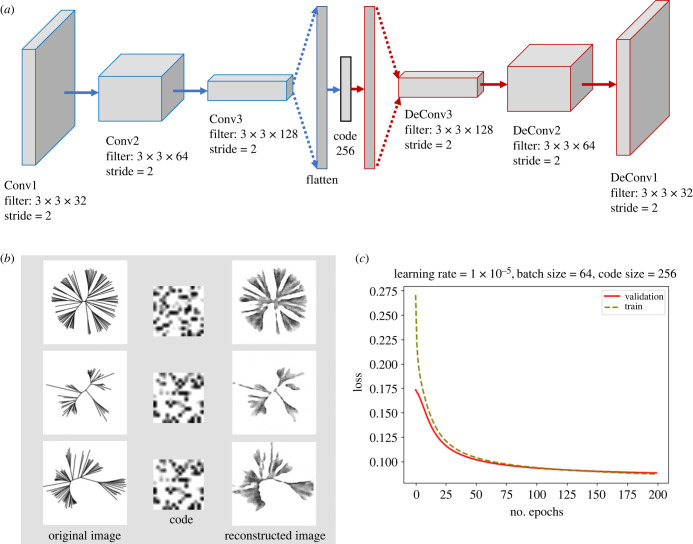
(*a*) The architecture of the CAE that we use to compress our images. The encoder consists of three convolutional layers followed by an embedded layer. The decoder consists of a fully connected layer and three convolutional transpose layers. (*b*) Some examples of the original images, compressed representation of the images (code) and the reconstructed images. (*c*) The average binary cross-entropy loss per epoch for training and validation steps. The title shows the parameters we used for training our model.

To reduce the dimensionality of our data (images, initially in 160^2^ = 25 600 dimensions), we use an auto-encoder. An auto-encoder is a neural network that is trained to create a low-dimensional representation of its input, from which it can reproduce that input (as its output). The network consists of two parts. The first part, called the encoder and denoted by *h* = *f*(*x*), maps the input *x* to a latent space *h* also called hidden layer or code. The second part, called the decoder and denoted by *r* = *g*(*h*), produces a reconstruction of the input from the latent space representation in the hidden layer *h* [21]. In our work, we use a convolutional auto-encoder (CAE). CAEs use convolutional layers in addition to fully connected layers. The advantages of using a CAE for image compression is that it can capture the most salient features of the images efficiently while preserving the local structure of the data and avoiding distortion of the feature space [22]. In designing the network, we use convolutional layers with stride (instead of convolutional layers followed by a pooling layer) in the encoder, and convolutional transpose layers with stride in the decoder, since the use of stride allows the network to learn spatial sub-sampling (up-sampling) from the data. For more details, see figure 1. The challenging part of designing an auto-encoder is determining the size of the embedding layer. Larger sizes would result in a network whose output is simply a copy of its input, defeating the purpose of an auto-encoder. Instead, we use *undercomplete* auto-encoders, whose hidden layer has a dimensionality considerably smaller than that of the input data, forcing the auto-encoder to learn its key features. We tried different code sizes to find the optimum one (see electronic supplementary material).

### (c) Clustering

We apply *K*-means clustering [23,24] with *K* = 3, affinity propagation [25,26] and hierarchical clustering [27,28] (also set to obtain K=3 clusters) to the low-dimensional representations *h* of our images. Affinity propagation uses a parameter called the ‘preference’, which influences the number of clusters; we tune this parameter manually to obtain *K* = 3 clusters, which results in a preference equal to −100. We then create a clustering graph with a node for each image and an edge between those pairs of images that are clustered together by all three methods. We use all sufficiently large connected components of this graph (those with at least 10 subtrees) as our final clusters. In other words, we create three partitions of the dataset, one per clustering algorithm, and compute the meeting point of these partitions.

### (d) Statistical analysis

Once the clusters have been identified, we carry out a statistical analysis to evaluate whether they can simply be explained by data characteristics such as sampling dates, sampling regions, sampling environments, or the bacterial species. Since the cluster *C* and sampling features *F* are categorical variables, we obtain a contingency table of *C* and *F*, and analyse the strength of association between them using three statistics. First, we use a corrected Cramér’s V (CCV) statistic, which is a *χ*^2^ statistic adjusted for the number of categories in *C* and *F*. The CCV takes on values in [0, 1], with values below 0.3 considered to indicate weak association [29]. We also report the 95% confidence interval on this statistic from the DescTools package [30]. We also use two clustering quality metrics, the adjusted Rand Index (ARI) and the normalized mutual information (NMI). The ARI is the fraction of pairs (*i*, *j*) of items assigned consistent values by *C* and *F* and adjusted for the chance expectation of this fraction; it takes on values bounded above by 1, with small or negative values indicating a chance association [31]. The NMI is the mutual information between *C* and *F*, i.e. the amount of information that the distribution of one conveys about the other, normalized by the maximum of the entropies of *C* and *F*; it lies in [0, 1], with values near 0 indicating a chance association, and is known to be a similarity metric [32]. These clustering quality metrics are computed using the aricode package [33].

We also evaluate the association of the clusters with a set *S* of common (continuous) tree shape statistics by carrying out a Welch’s *t*-test [34] from the stats package [35] for every statistic and pair of clusters, performing a Bonferroni multiple testing correction [36] for a hypothesis space of size $N=\left(\begin{array}{c} \left|C\right|\\ 2 \end{array}\right)\times |S|$, i.e. the number of cluster pairs times the number of statistics, meaning that only tests with a *p*-value below 0.05/*N* are considered to be statistically significant.

## 3. Results

The CAE that produces the best performance in the dimensionality reduction of our images has the following architecture: the encoder consists of three convolutional layers followed by an embedded layer (fully connected layer). The optimal code size in this network has 256 units. The decoder consists of a fully connected layer and three convolutional transpose layers. The trained network efficiently encodes the original images of size 160 × 160 into feature space arrays of size 16 × 16. For more details on the structure of the CAE and the parameters that we used, see figure 1 and the electronic supplementary material. We focus on the non-overlapping subtrees as they create a clearer clustering structure.

**Figure 2.**
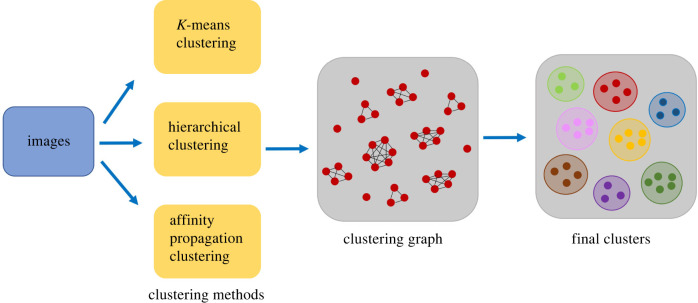
Our approach for combining three clustering algorithms using a graph-based method. In the intermediate clustering graph, the cliques determine the final clusters.

**Figure 3.**
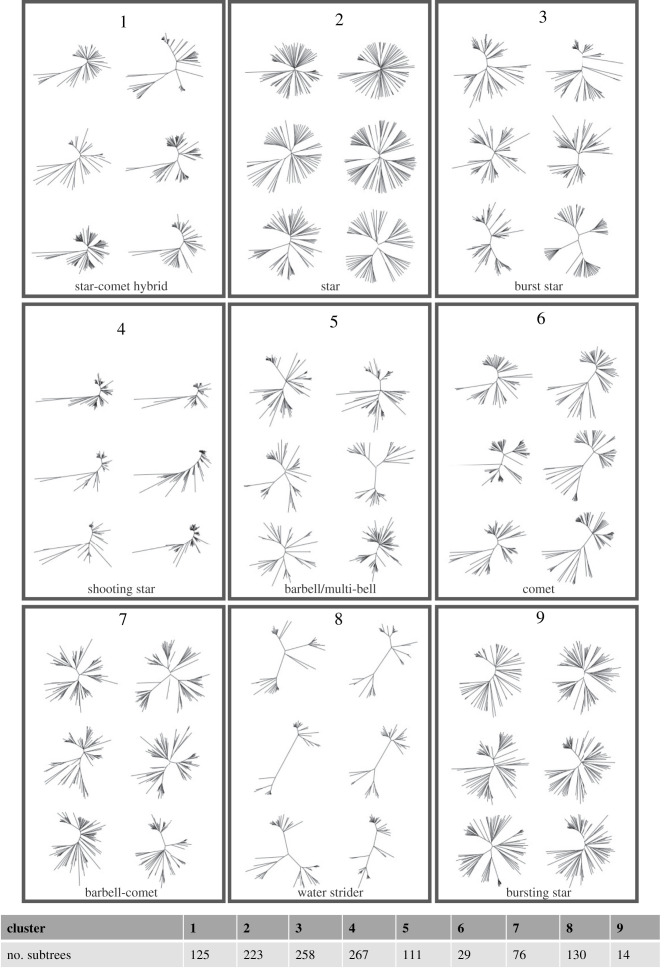
Representatives of the nine clusters resulting from the graph-based clustering method (we randomly chose six images from each cluster). They include familiar shapes such as comets and star phylogenies; we have named the other clusters, but there is a range of increasing population structure moving from the star phylogenies (cluster 2; relatively little separation between distinct sub-populations) to the ‘water striders’ (cluster 8 strong separation). Clusters 3, 5, 7 and 9 are along this continuum. The bottom panel shows the numbers of subtrees in each cluster.

The resulting clustering graph (figure 2) has 16 connected components with sizes ranging from 1 to 267. We use the components of size at least 10 as our final clusters, which results in nine clusters. We find that 98% (1233/1254) of the original images are in these nine clusters. Figure 3 shows six trees randomly chosen from each cluster. Electronic supplementary material, figure S3 also shows the central representatives of the clusters. The complete set of tree images from each cluster are available at https://github.com/MarHayat/TreeShapeVAE. Cluster 2 is a star phylogeny, showing relatively little population structure. This can reflect high levels of recombination, disrupting population structure, or rapid diversification without much selection. Cluster 6 shows the classic ‘comet’ shape, with lower-diversity relatively unstructured ‘heads’ in the context of more diverse ‘tails’. We have dubbed cluster 4 the ‘shooting star’ shape, which has similarities to the comet. These have lower-diversity, here tightly grouped sets of taxa at one end of a more linear tree shape, and longer edges capturing comparatively diverse populations. The shape arises because there are more taxa in the less-diverse tight groups typically appearing on the top right. Clusters 3, 5, 7 and 9 reflect a continuum of population structure, moving from the star phylogeny (long and relatively similar terminal branches and relatively short internal branches) to more ‘barbell’ shapes illustrating two or more distinct populations separated by longer internal branches. The sub-populations themselves have limited additional structure in clusters 3 and 9, but have more in clusters 5 and 7. The corresponding shapes are less circular as a result. Finally, we have dubbed cluster 8 the ‘water striders’; these are at the other end of the population structure spectrum from the star phylogeny, with long internal branches separating very distinct sub-populations. We note that the clusters do share elements, and some trees in cluster 4 are similar to the comets in cluster 6, and so on. Furthermore, we have named the clusters for reference and to begin to characterize the kinds of trees we have found, but we recognize that these names are somewhat *ad hoc*. Images of the trees in each cluster can be found at https://github.com/MarHayat/TreeShapeVAE/blob/main/Clusters.zip.

**Figure 4.**
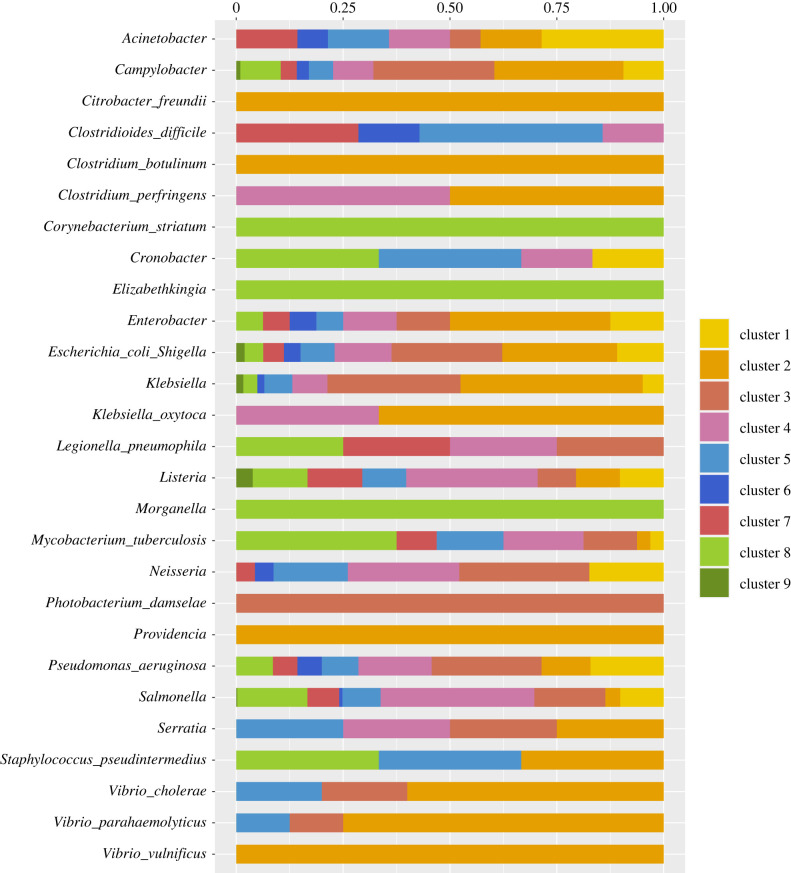
This figure shows the distribution of the species across the clusters. Some species (*Citrobacter freundii* (3), *Clostridium botulinum* (4), *Corynebacterium striatum* (1) and *Vibrio vulnificus* (22)) as well as genera (*Elizabethkingia* (15), *Morganella* (6) and *Providencia* (7)) are found in a single cluster (C2 or C8). Numbers of trees are in parentheses; except for *Vibrio vulnificus* species whose trees are in a single cluster simply have few subtrees in the data—see table 1.

Figure 4 shows the distribution of the species across the clusters. The subtrees of some species including *Elizabethkingia* are entirely in a single cluster (cluster 8), though these are predominantly species for which there are very few subtrees (table 1). A few species including *Salmonella* are distributed among all the nine clusters, probably because these are groups with large numbers of taxa and subtrees in the data. Some of the species, such as *Serratia*, are distributed equally among several clusters. Overall, the cross-tabulation of species by cluster has a CCV statistic of 0.16 (0.14–0.18), indicating a weak association. Similarly, the ARI of 0.054 and the NMI of 0.095 indicate that the distribution of species across the clusters is consistent with chance allocation.

**Figure 5.**
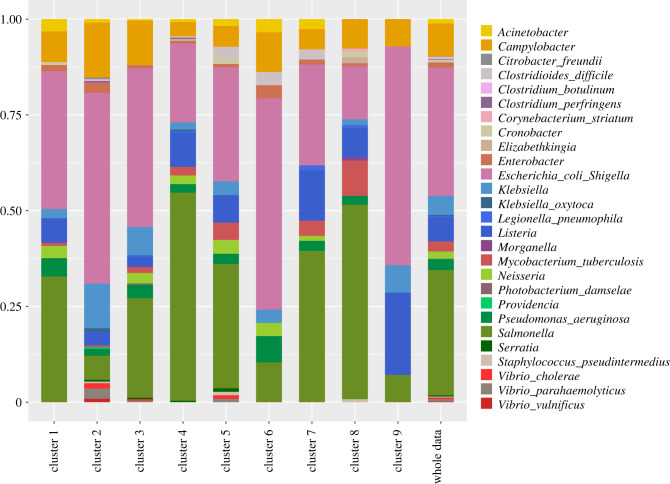
The species composition of each cluster.

Figure 5 shows the composition of each cluster. Cluster 2 has fewer *P. aeruginosa* trees than the dataset at large; cluster 6 has fewer *Listeria* (cluster 9 has more) and cluster 8 has fewer *E. coli* than the dataset at large. However, we do not have a clear interpretation for these results because the presence or absence of a particular shape is not just determined by the species, but by the sampling process, the species’ ecology and epidemiology, and its overall population structure.

**Figure 6.**
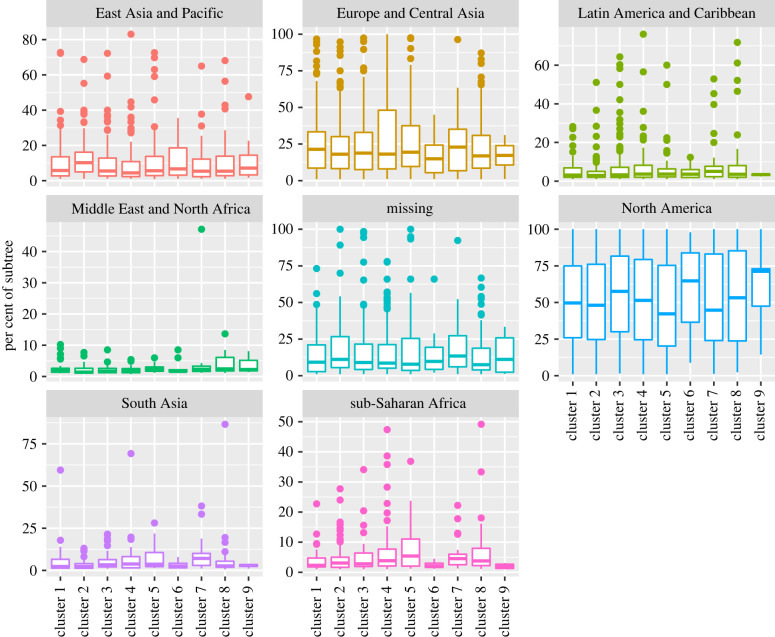
Percentages of the tips in a subtree in each geographical region, by cluster.

**Figure 7.**
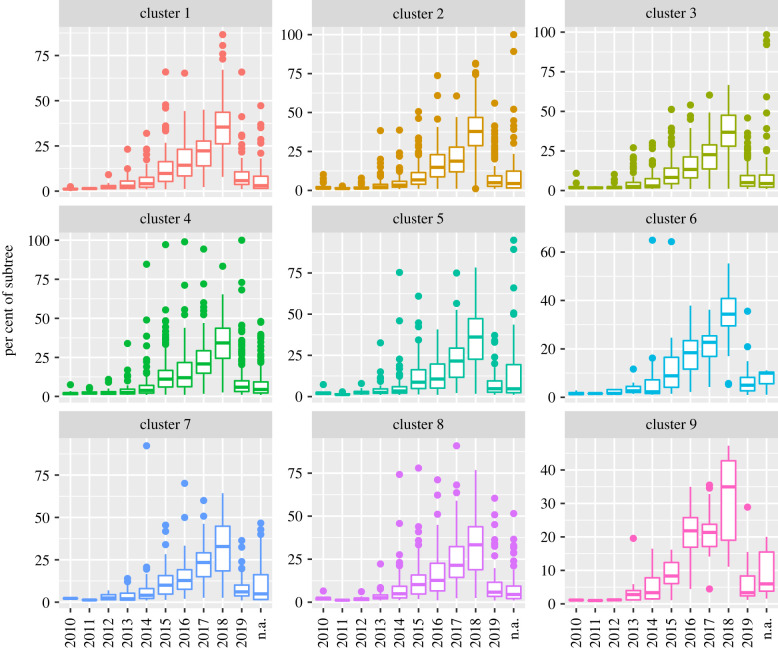
Percentages of the tips in subtrees in each year, organized by cluster.

We also visualize the geographical distributions of the species on each cluster in figure 6. This plot reveals that our data originate more in North America, Europe and central Asia than other regions. It also shows that species are approximately equally distributed among the clusters from the geographical perspective except for cluster 9, which is by far the smallest of the clusters. This approximated independence of clusters by region is corroborated by a CCV of 0.046 (0.043–0.048), an ARI of 0.001 and an NMI of 0.004, indicating a very week association between clusters and region. The distribution of the dates of the species on each cluster is shown in figure 7. As shown in this figure, the dates are also distributed approximately equally among the clusters, as also evidenced by a CCV of 0.031 (0.029–0.033), an ARI of 0.003 and an NMI of 0.002.

**Figure 8.**
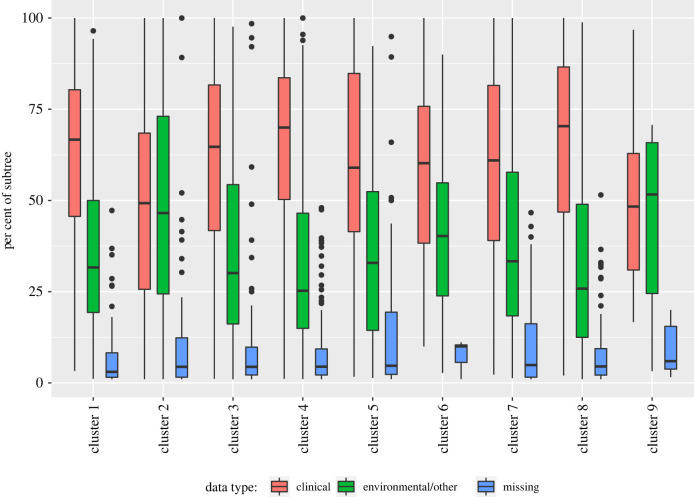
Subtree composition by epidemiological origin, organized by cluster. Overall, there are 49955 samples (60%) tagged as clinical, 30252 (36%) labelled environmental/other and 3708 (4%) with this information missing. (Online version in colour.)

The distribution of the subtrees in each cluster by epidemiological origin, namely, clinical, environmental or unspecified, is shown in figure 8. As for the other characteristics, the distribution of the origin does not vary significantly between clusters. The CCV value of 0.092 (0.087–0.097), the ARI of 0.004 and the NMI of 0.004 suggest that the distribution of the epidemiological origin is not significantly different from one that is independent of cluster. Overall, this suggests that the clusters capture subtree variation that is not accounted for by simple sampling biases, whether temporal, regional, contextual or species-specific. However, there are known biological origins for the kinds of images we have found, in particular, the comet and star images (e.g. adaptation followed by rapid diversification from a common origin). Such evolutionary processes can happen in any year or part of the world, and can occur in human or environmental settings.

**Figure 9.**
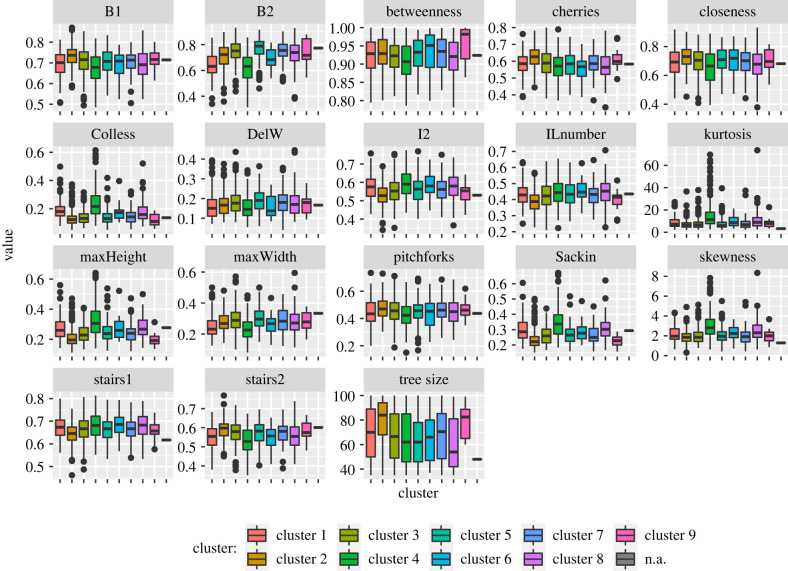
Normalized tree shape statistics for the nine clusters.

We also compared tree shape statistics and our clustering patterns. For this purpose, we computed a set of tree shape statistics [37,38] for the trees on each cluster. We computed both unnormalized (electronic supplementary material, figure S2) as well as normalized versions (figure 9) of each statistic, with the exception of those that are intrinsically normalized. See electronic supplementary material, table S1 for the details of the tree shape statistics and the normalizations. Figure 9 shows the tree shape statistics.

We begin by noting that among all the statistics, the tree size itself is only able to distinguish between 6 out of $\left(\begin{array}{c} 9\\ 2 \end{array}\right)=36$ pairs of clusters, meaning that very few cluster pairs have mean tree size differences that are statistically significant according to Welch’s *t*-test with Bonferroni correction. Nevertheless, we prefer to focus on the normalized statistics, which take into account (albeit imperfectly) the distribution of the statistic among all trees of the same size, by dividing the results by their maximum possible value. We find that the Sackin imbalance is able to distinguish between 18 out of 36 cluster pairs, while the Colless imbalance, B2, and maxHeight distinguish between 15, 14 and 14 out of the 36 pairs, respectively. None of the other statistics can distinguish more than 10 pairs of clusters. Overall, 13 of the 36 pairs of clusters cannot be discriminated by any of the statistics individually while another 5 of the pairs can only be discriminated by 1 statistic each. The most challenging clusters to differentiate using shape statistics alone are clusters 6, 7 and 9, which happen to be the three smallest clusters, with shape statistics being able to differentiate each of them from only 3 of the other clusters. This result is in general agreement with the findings in [39,40] which evaluate the power of tree shape statistics in distinguishing between different tree shapes.

## 4. Discussion

Phylogenetic trees are a powerful tool to visualize biological diversity and evolution, showing relatedness patterns among a set of organisms. Tree images are a familiar graphical description of microbial evolution and are a convenient way to place new WGS data into an interpretable context. We clustered tree images extracted from large-scale bacterial phylogenetic trees. We found nine major groups of tree images, including ‘comet’, ‘star’ and ‘barbell’ phylogenies, and commented on the evolutionary and epidemiological scenarios that these shapes illustrate. These human-friendly graphical descriptions of trees may become convenient ways for researchers to place new WGS datasets within an interpretable context, for example ‘this new isolate is in the tail of a comet in this organism’.

Our key interest is in uncovering what kinds of trees are present in bacterial genomic data. We take a ‘natural history’ approach, using an exploratory analysis of a large number of phylogenetic trees from wide-ranging pathogenic bacterial species to group and catalogue trees. In this endeavour, we have aimed to use a qualitative piece of information—the tree image—as a unit of analysis, informed by interpretations of the MRSA emergence ‘comet’ and by what we see as several advantages of radial tree images. The radial style of tree visualization is relatively robust to the sampling density and tree size, within reason. For example, a ‘star phylogeny’ (cluster 2) will have a similar radial shape whether it has 50 tips or 500 tips, and the radial layout ensures that the visualization of the tree will attempt to fill the angles around the tree’s centre. For these reasons, we did not use circular or rectangular layouts, though these are more commonly used when illustrating taxon-level metadata alongside phylogenies. The comet, and other shapes, are not likely to be consistently recognizable as such in different layouts. We considered using tree statistics (imbalance and so on) for clustering, but these depend strongly on the number of tips and there is no ready interpretation for the linear combinations of tree statistics that would emerge as characteristic of clusters (if derived from those statistics). We also considered using the graph structure (nodes and edges) of the tree, but there are many ways to write an edge list that define the same graph (or tree), and as with tree shape statistics, these representations will depend strongly on the tree size. Most fundamentally, though, trees and the tree visualizations we have used capture relatedness at the level of a *collection* of taxa. Using images also allows us to leverage deep learning tools, which are powerful and are optimized to analyse images.

While the shapes in our images are relatively robust to tree size and therefore to random uniform sampling, they are not always robust to biased sampling (although they are relatively robust to multiple observations of nearly identical taxa). This is true of any analysis of phylogenetic trees, but large compilations of data from multiple studies have an additional vulnerability as the sampling approach is likely to be highly variable across the dataset (unlike in a single study where it is understood and/or chosen by the study authors). Our dataset is large and ranges widely, and it is unlikely that sampling biases (also likely to be diverse) drive the results we have obtained. However, the clusters we identify reflect the clusters in phylogenetic trees from pathogens on NCBI, and not necessarily the patterns in the phylogenetic trees in bacteria, or pathogenic bacteria, in general. An apparent ‘comet shape’ could be obtained by compiling focused and sequencing-intensive studies sampling a particular strain or complex that has diversified relatively recently, together with less intensive surveillance collecting additional ‘background’ isolates. Or, as with MRSA, the apparent expansion could be due to selection, adaptation and rapid dissemination. A research and sampling focus may occur precisely because of a phenomenon of interest such as antimicrobial resistance, unusually large outbreaks, increased pathogenicity or increased virulence.

Linking ‘metadata’ to the taxa in large databases like NCBI would help to elucidate shared evolutionary, ecological and epidemiological processes giving rise to similar trees. It would also help to distinguish among distinct processes that may give rise to similar trees, for example distinguishing rapid growth from an increase in sampling. While in many studies phylogenies are used to illustrate the extent to which metadata fields such as geography or antibiotic resistance are grouped according to shared ancestry, here we would seek to determine how the phylogenies group according to the metadata. Particular helpful metadata would include the reason for isolate collection, the reason for sequencing, a link to a relevant study and study design, information about antimicrobial resistance, pathogenicity/virulence, information about recombination and other relevant context. These would add a tremendous amount of value to large compilations of sequence data (and phylogenetic trees).

Our method can be applied to phylogenetic trees reconstructed from different species and organisms. Clustering tree images reconstructed from viruses such as influenza virus, HIV and SARS-CoV-2, and seeing what tree images occur in different phylogenetic trees of viruses, could similarly illustrate a range of evolutionary patterns. A comparison between the clustering of bacterial tree images and the clustering of viral tree images might reveal insights about different evolutionary scenarios between viruses and bacteria. However, the largest advances will likely be through linking data to inform sampling, selection, ecology and epidemiology to these large-scale analyses; this will enable both improved interpretation and hypothesis generation and testing. All the tree images and codes are available at https://github.com/MarHayat/TreeShapeVAE.

## Data Availability

The data are provided in electronic supplementary material [41].
